# Selective interactions between diverse STEs organize the ANT-C Hox cluster

**DOI:** 10.1038/s41598-018-33588-4

**Published:** 2018-10-11

**Authors:** Mo Li, Zhibo Ma, Sharmila Roy, Sapna K. Patel, Derrick C. Lane, Carly R. Duffy, Haini N. Cai

**Affiliations:** Department of Cellular Biology, University of Georgia, Athens, GA 30602 USA

## Abstract

The three-dimensional organization of the eukaryotic genome is important for its structure and function. Recent studies indicate that hierarchies of chromatin loops underlie important aspects of both genomic organization and gene regulation. Looping between insulator or boundary elements interferes with enhancer-promoter communications and limits the spread active or repressive organized chromatin. We have used the SF1 insulator in the *Drosophila* Antennapedia homeotic gene complex (ANT-C) as a model to study the mechanism and regulation of chromatin looping events. We reported previously that SF1 tethers a transient chromatin loop in the early embryo that insulates the Hox gene *Sex comb reduce* from the neighbor non-Hox gene *fushi tarazu* for their independent regulation. To further probe the functional range and connectivity of SF1, we used high-resolution chromosomal conformation capture (3C) to search for SF1 looping partners across ANT-C. We report here the identification of three distal SF1 Tether Elements (STEs) located in the *labial*, *Deformed* and *Antennapedia* Hox gene regions, extending the range of SF1 looping network to the entire complex. These novel STEs are bound by four different combinations of insulator proteins and exhibit distinct behaviors in enhancer block, enhancer-bypass and boundary functions. Significantly, the six STEs we identified so far map to all but one of the major boundaries between repressive and active histone domains, underlining the functional relevance of these long-range chromatin loops in organizing the Hox complex. Importantly, SF1 selectively captured with only 5 STEs out of ~20 sites that display similar insulator binding profiles, indicating that presence of insulator proteins alone is not sufficient to determine looping events. These findings suggest that selective interaction among diverse STE insulators organize the *Drosophila* Hox genes in the 3D nuclear space.

## Introduction

The three-dimensional (3D) organization of the genome is critical for its function including transcription regulation^1–8^. Recent studies indicate that extensive loop structures underpin the genomic architecture in mammals and *Drosophila*^3,5,9–14^. Chromatin boundaries, also known as insulators, are DNA-protein complexes originally known to separate and insulate neighboring chromatin domains. Interactions between insulator sites tether chromatin loops, which can block or promote enhancer-promoter interactions^15–22^. These loops can also impede the spread of silent or active chromatin^22–25^. Multiple classes of insulator complexes, represented by SuHw, dCTCF, GAF and BEAF proteins, are known to facilitate chromatin looping in *Drosophila*^14,17,26–34^. Genome-wide distributions of these insulator complexes are partially overlapping and the functions of the loops they tether are largely unknown. Mounting evidence suggests that interactions between insulators can be tissue- and developmental stage-specific, providing a potential mechanism for developmental gene regulation^22,32,35–38^. However, the organization and regulation of chromatin loops, especially defined at high resolution and in the context of animal development, remain poorly understood.

**Figure 1:**
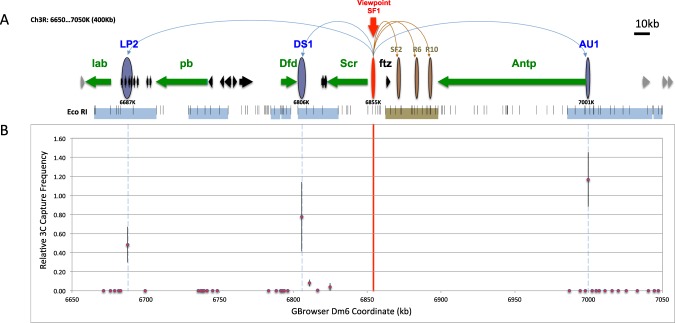
Chromosome Conformation Capture (3C) identification of novel SF1-Tethering-Elements (STEs) in ANT-C. (**A**) Diagram of ANT-C. Map coordinate is based on the *Drosophila* genome browser dm6 (BDGP R6 Plus ISO1 MT)^92^. Horizontal arrows indicate genes and their transcription direction. Green arrows represent homeotic genes; black and gray arrows represent non-homeotic genes within and outside of ANT-C, respectively. The horizontal bars below genes represent regions tested in the SF1-based 3C captures in the current study (light blue) and a previous study (brown)^22^. Small vertical lines represent EcoR I sites. Vertical ovals indicate SF1 (red) and STEs identified in the current study (blue) and those from previous work (brown)^22^. Gbrowser coordinates for the center of the captured fragment is labeled underneath the blue oval. Long curves on top represent looping interactions between SF1 and STEs. (**B**) Relative SF1-capture frequency of 39 EcoR I fragments in the ANT-C intergenic regions. The coordinates at the center of each fragment is plotted against the average capture frequency over control (for detail see methods)^22^. Red vertical line represents the position of SF1 and blue dashed lines represent the positions of STEs.

The *Drosophila* Homeotic/Hox genes are activated in a tissue-specific pattern by numerous enhancers during early embryonic development. Their transcriptional status are then maintained by epigenetic mechanisms mediated by the Polycomb Group (PcG) and Trithorax Group (Trx-G) complexes in late development^39–43^. As both enhancer- and chromatin-mediated transcriptional regulation are influences by the formation of chromatin loops, the homeotic/Hox complexes provide a good model for elucidating the mechanism and regulation of genomic loops. We have previously identified a hub of chromatin loops anchored by the SF1 insulator in the *Drosophila* Antennapedia homeotic complex (ANT-C, Fig. 1A)^22^. SF1, located in the regulatory region of the *Sex comb reduced (Scr)* Hox gene, tethers with the SF2 insulator during early embryogenesis (Fig. 1A)^22^. This creates a chromatin loop that not only encloses and separates the neighbor non-Hox gene *fushi tarazu* (*ftz)* from the surrounding Hox genes, but also reconnects the interrupted *Scr* regulatory region and facilitates *Scr* distal enhancers (Fig. 1A)^22,34,44–50^. We showed that the transient loop correlates, both in genomic extent and timing of formation, with an active chromatin domain surrounding the *ftz* transcription unit. The loop also coincides with a reduced access between the *ftz* gene and the neighboring Hox enhancers^22^. We further showed that SF1 also contacts several other local regions between *Scr* enhancers and the *Scr* Polycomb Response Elements (PREs) in mid and late embryogenesis, possibly regulating their access to the *Scr* promoter. Our results suggest that formation of chromatin loops can be a developmentally regulated and play important roles in gene regulation.

These findings raised several questions regarding how ANT-C is organized. For example, is SF1 only a local chromatin organizer within the *Scr-ftz* interval? How many major units, as defined by independent loops are there in ANT-C? Alternatively, can SF1 tether long-range chromatin loops beyond the *Scr-ftz* region? How many different subclasses of tether-elements can SF1 contact? What are the unique functions and *cis-* and *trans*-components of other potential STEs? In the current study we begin to address these questions by scanning for SF1-contacting points outside of the *Scr-ftz* region within ANT-C. Using chromosome conformation capture (3C) we identified three novel STEs near the *labial (lab)*, *Deformed (Dfd)* and *Antennapedia (Antp)* Hox genes. The three STEs span the length of ANT-C, ranging from 50 to 167 kb distance from SF1. These findings suggest that SF1 is part of a larger network of chromatin loops that organize the ANT-C Hox cluster. We report that at least four different combinations of insulator proteins associate with these novel STEs, which exhibit distinct behaviors in enhancer block, enhancer bypass and boundary functions. Importantly, SF1 selectively captured with five STEs out of 17 sites that exhibit similar binding profiles for insulator proteins, suggesting that binding by insulator protein alone is insufficient to determine looping events. Further, SF1 and STEs collocalize with all but one of the major domain boundaries between repressive chromatin domains around Hox genes and the less repressive domains around non Hox domains, underscoring the functional relevance of these looping events in organizing the ANT-C Hox complex in *Drosophila* embryos.

## Results

### Identification of novel SF1-tethering elements (STE’s) in the Antennapedia homeotic gene complex

To probe the functional range of SF1, we searched major ANT-C intergenic regions outside of the *Scr-ftz* interval for DNA regions that interact with SF1. To this end, we tested SF1 capture of 39 EcoRI fragments using high-resolution chromosomal conformation capture (3C) in 0–20 hour old embryos (Fig. 1A, see Methods)^20,51–54^. These elements cover most of the non-transcribed regions in ANT-C (blue horizontal bars, Fig. 1A) outside the *Scr-ftz* interval (brown horizontal bar, Fig. 1A), which was described previously^22^. Three elements preferentially interact with SF1 (Figs 1B, S1A–C, blue ovals, Fig. 1A). Two of these, LP2 and AU1, are located near the *lab* and *Antp* genes at 184 kb and 146 kb from SF1, respectively. They capture strongly and selectively with SF1, while the surrounding regions exhibited no capture (Figs 1B, S1A,C). This is consistent with previous observations that non-specific captures due to genomic linkage is at a minimum at these distances^51,55^. DS1, the third region captured by SF1, is located immediately downstream of the *Dfd* and *Scr* transcription units, approximately 50 kb from SF1 (Figs 1B, S1B). Since genomic elements within such distances are known to capture at relatively high frequencies, we applied a distance-frequency reference curve for the *Drosophila* genome to evaluate the significance of capture (dotted curve, Fig. S1B)^22^. Of the four fragments in the *Dfd-Scr* intergenic region, the EcoRI element at 6806 K captured with SF1 at a frequency significantly above the expected value (P < 0.0016), whereas the other three fragments captured SF1 did not. Negative controls without cross-linking or without ligase yielded no capture products as expected (not shown). The identification of these novel STEs in the distal regions of ANT-C extends the SF1 contact range beyond the local *Scr-ftz* region, revealing a long-range chromatin loop network that could organize the entire Hox cluster.

### Novel STEs colocalize with chromatin boundaries that separate distinct histone modification domains

**Figure 2:**
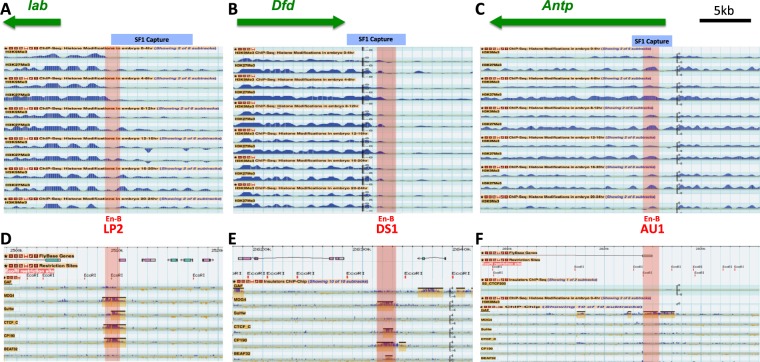
Novel STEs demarcate chromatin domain boundaries and bind to insulator proteins. Top, screen crops of the repressive H3K9Me3 and H3K27Me3 ChIP-seq profiles surrounding STEs in *lab* (**A**), *Dfd* (**B**), and *Antp* (**C**) genomic regions from different embryonic stages. The EcoRI fragments captured by SF1 are indicated by the blue-shaded horizontal bars on top. The sub-fragments containing enhancer-blocking activities (En-B, also see Fig. 3) are indicated by the red shaded vertical bars with their names indicated below. Bottom, screen captures of ChIP-Chip profiles of known insulator proteins surrounding the STEs in 0–12 h embryos^64^. Yellow-shaded boxes represent called peaks for bound proteins. Map coordinate is based on ModEncode GBrowser dm3 (BDGP R5).

Insulator-tethered chromatin loops are known to impede the spread of silent or active chromatin and therefore often correspond to boundaries between domains of distinct histone modification marks^22,23,56–60^. For example, SF1 and its local partner SF2 flank an active chromatin domain that encloses the *ftz* gene and separate it from the surrounding silent chromatin in the early *Drosophila* embryo^22^. To probe whether the newly identified STEs correspond to such chromatin domain boundaries, we examined the histone modification profiles surrounding these elements in the modENCODE database^61–63^. We found that the STEs in the *lab* and *Dfd* regions indeed colocalize with transition points between active and repressive chromatin in the *Drosophila* embryos (Fig. 2A,B). The ~8-kb *lab* STE is located ~5 kb upstream of the *lab* promoter, separating it from multiple small non-Hox genes (Fig. 2A). The ~5.5-kb *Dfd* STE is located ~3 kb downstream of the *Dfd* gene, separating it from a series small tRNA and non-coding RNA genes (Fig. 2B). In both cases, the STEs (blue shaded bar on top) contain the border between the Hox gene domains with higher levels of in repressive H3K9me3 and H3K27me3 marks, and the non-Hox gene regions that are depleted of these marks (Fig. 2A,B). In contrast to these two STEs, the *Antp* STE does not clearly demarcate a boundary between different histone modification domains. The repressive histone marks appear to be enriched to a comparable level on both sides of the STE (Fig. 2C). It is possible that the *Antp* STE separate distinct chromatin domains only in selected tissues. It is also possible that loops formed at the *Antp* STE provide a different genomic or regulatory function.

To understand the different boundary behaviors exhibited by these new STEs, we further examined their binding profiles by known insulator/boundary proteins in the *Drosophila* embryo using the ModENCODE chromatin immunoprecipitation (ChIP) database^61,63,64^. All three new STEs are bound by insulator/boundary proteins, although each with a different combination. For example, both *lab* and *Dfd* STEs are bound by dCTCF, CP190, and Mod (mdg4) (Fig. 2D,E). However, the *lab* STE is also bound by SuHw, whereas the *Dfd* STE is not. In comparison, the *Antp* STE is bound only by GAF, a zinc finger/BTB domain insulator protein that also plays a role in transcription activation and PcG and TrxG mediated functions (Fig. 2F)^65–71^. The difference in the insulator complexes assembled on these STEs may determine their boundary/barrier behavior. The binding profiles of the new distal STEs are reminiscent of those of SF1 and its local STEs (Fig. S1)^22^. For example, SF1 and its local partner SF2 are bound by dCTCF, CP190 and Mod (mdg4). Although SF1 does not directly bind to GAF, R10, an STE located at the end of the *Antp* transcription unit, binds strongly to GAF. These findings suggest that SF1 can contact genomic loci bound by different insulator complexes and possibly serve different functions.

### Novel STEs contain enhancer-blocking activities in transgenic *Drosophila*

**Figure 3:**
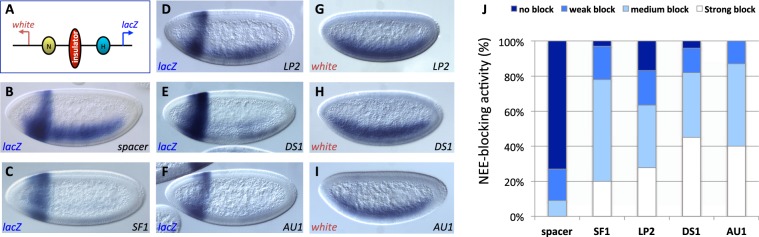
STEs exhibit diverse enhancer-blocking activity in transgenic *Drosophila*. (**A**) Diagram of the pWNHZ-STE transgene vector containing divergently transcribed *lacZ (*blue arrow) and *miniwhite* (mw, red arrow) reporters flanking the NEE (yellow circle) and H1 (blue circle) enhancers (see Methods). Red vertical oval: STEs or spacer control inserted between NEE and H1. (**B**–**J**) Representative images of transgenic embryos after whole mount *in situ* hybridized with the anti-*lacZ* (**B**–**F**), or the anti-*white* (**G**–**I**) RNA probes. DNA elements in these embryos are labeled at bottom right of each photo and probes used on bottom left. Embryos are shown in sagittal views with anterior to the left and dorsal up. (**J**) Quantitation of NEE-blocking in the whole neuroectoderm in transgenic embryos stained with the anti-*lacZ* probe (see Methods for details).

An important function of chromatin insulators is to modulate enhancer-promoter communications^16,47,72–77^. The new STEs are located near Hox regulatory elements and may be involved in organizing enhancer traffic. We tested new STEs for their insulator function using an enhancer-blocking assay in transgenic *Drosophila* embryos^22,34,78–80^. The assay transgene contains divergently transcribed *white* and *lacZ* reporters driven by two tissue-specific enhancers, the Neuroectoderm Enhancer (NEE) and the Hairy Stripe 1 enhancer (H1, Fig. 3A). Known insulators such as SF1 but not a neutral spacer, when inserted between the two enhancers, can block the distal NEE from the *lacZ* reporter, reducing its expression in the horizontal neuroectoderm, without affecting the H1 driven *lacZ* expression in the head (Fig. 3B,C, see quantitation in Fig. 3J)^22,34,78–80^. We first dissected the ~9-kb *lab* STE in the transgenic insulator assay. The proximal end of the EcoRI fragment overlaps with ChIP-seq peaks for multiple insulator proteins (Fig. 2D). We tested a ~1.4-kb fragment, named LP2, that includes the major peaks of insulator binding and found it to contain significant enhancer-blocking activity (Fig. 3D, also see quantitation in Fig. 3J). To confirm that the loss of the *lacZ* expression is not due to a silencing effect from the insulator, we also examined the divergently transcribed mini-*white* reporter. We found a reduction of H1-driven *white* expression in the head but not NEE-driven *white* expression in the ventral lateral region (Fig. 3G). This result indicates that LP2 contains enhancer-blocking/insulator activity. We then examined the STE fragments from the *Dfd* and *Antp* regions. Each of these regions contains a single enhancer-blocking sub-fragment, named DS1 and AU1, respectively (Figs 2B and 3E–J). In both cases, the enhancer-blocking fragment colocalizes with the DNA region bound by insulator proteins (Fig. 2E,F). Our enhancer-blocking results indicate that all three distal STEs function as autonomous insulators, independent of their native genomic context and away from SF1. It is possible that the STEs tether chromatin loops with insulator complexes near the insertion sites to block transgenic enhancers. Previous studies indicate that enhancer-blocking and chromatin-blocking function of a boundary element may be mediated by distinct mechanisms and separable^29,76,77^. The differential boundary behaviors exhibited by the STEs further suggest that these activities could be provided by different insulator complexes.

### SF1-STE pairing can facilitate enhancer-bypass

**Figure 4:**
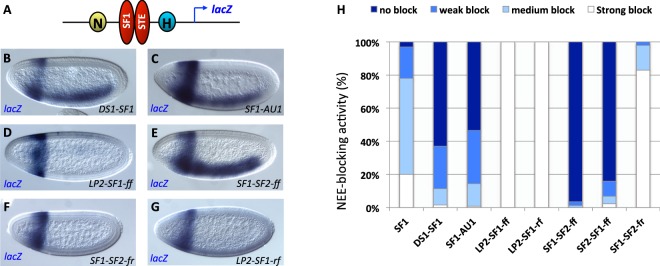
SF1-STE pairing mediates enhancer bypass. (**A**) Diagram of the enhancer-bypass transgene containing SF1and STE placed in tandem between NEE and H1 in the pWNHZ vector (red ovals, also see Methods). (**B**–**G**) Representative images of transgenic embryos after whole mount *in situ* hybridization with anti-*lacZ* probes. DNA elements in these embryos are labeled at bottom right of each photo and probes used on bottom left. Orientation of the elements relative to the endogenous arrangement is indicated by “f” (forward) or “r” (reverse). (**H**) Quantitation of NEE-blocking in by tandem insulators in bypass transgenic embryos (see Methods for details).

We further tested whether the enhancer-blocking activity of the new STEs is cancelled by their tandem arrangement with SF1. Tandem pairing between certain insulators are known to neutralize their enhancer-blocking function^16,47^. This “enhancer bypass” phenomenon suggests that the formation of chromatin loops is necessary for enhancer blocking^16,47,71,81–86^. It is also an indication that the two insulators interact with each other directly. To this end, each of the new STEs was placed in tandem with SF1 and inserted between the NEE and the H1 enhancers (Fig. 4A). Transgenic embryos were examined for blocking of NEE enhancer on the *lacZ* reporter gene. For AU1 and DS1, we observed a strong recovery of NEE-driven *lacZ* expression in transgenic embryos, suggesting that pairing of SF1 and these two STEs neutralized their enhancer-blocking function (Fig. 4B,C,H, compared to Fig. 3E,F,J). However, we observed no enhancer-bypass with the LP2-SF1 pairing (Fig. 4D,H). We have recently found that for certain SF1-STE pairings, enhancer-bypass occurs in an orientation-dependent fashion. For example, SF1-SF2 pairing in a forward-forward (SF1-SF2-ff or SF2-SF1-ff) arrangement was shown to mediate enhancer-bypass (Fig. 4E,H)^22^. However, when SF2 is placed in an inverted orientation relative to SF1 (SF1-SF2-fr), bypass was not observed (Fig. 4F,H). In fact, an augmentation of insulator activity beyond that of SF1 or SF2 alone was observed (Figs 3J and 4H)^22^. This result suggests that formation of chromatin loops by insulator pairing may be affected by the relative orientation of the DNA sequences and/or protein complexes. Based on these findings, we also tested LP2-SF1 pairing in opposite orientation (LP2-SF1-rf). As shown in Fig. 4, inverting LP2 did not lead to enhancer-bypass, but rather an augmentation of NEE block (Fig. 4F,J, compare to Fig. 3D,J). The inability to cancel with SF1 might be due to the unique insulator complexes bound at LP2. Although LP2, as SF2 and DS1, is bound by dCTCF, CP190 and Mod (mdg4), it is also bound by SuHw whereas SF2 and DS1 are not. This difference might contribute to its inability to cancel with SF1. Certain insulators are known to not bypass when placed in tandem and this was attributed to their not interacting with each other *in vivo*^77^. Since LP2 was identified through its capture with SF1, it is possible that SF1 and LP2 do not directly interact with each other, and that their capture in 3C could be mediated by mutual partners.

## Discussion

**Figure 5:**
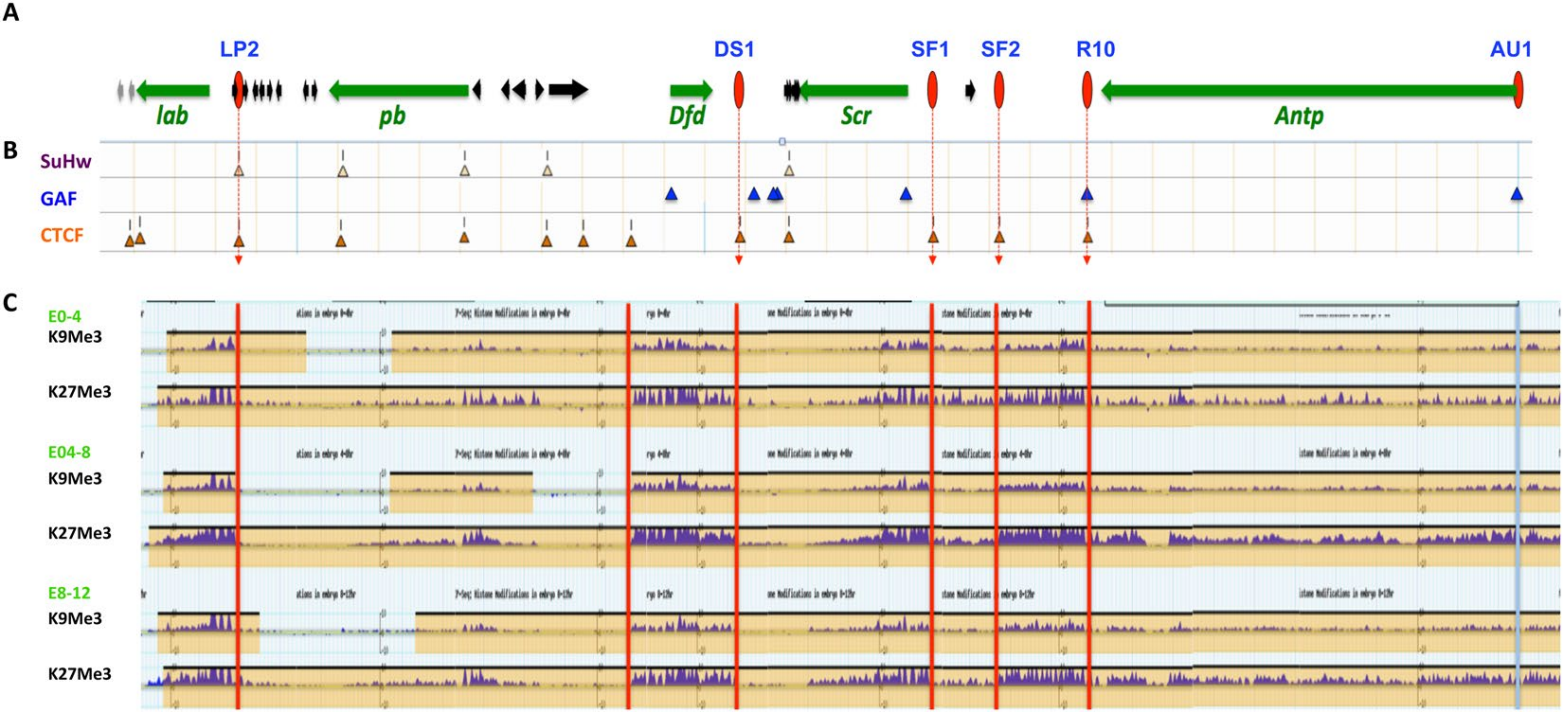
Selective interactions between diverse STEs organize the ANT-C Hox cluster. (**A**) ANT-C genomic map. Horizontal arrows represent Hox **(**green) and non-Hox (black and grey) genes with gene names below. Red ovals and red dash lines indicate SF1 and STE insulators. (**B**) Distribution of major insulator proteins across ANT-C. ChIP-seq peaks of SuHw (class II), GAF, and dCTCF (class I) insulator proteins are indicated by yellow, blue and orange triangles, respectively^64,92^. (**C**) Repressive histone domains in ANT-C during *Drosophila* development. Screen crop of ChIP-seq profiles of H3K9Me3 an H3K27Me3 in 0–4, 4–8 and 8–12 hour old *Drosophila* embryos (http://www.genome.gov/modENCODE). Red vertical lines indicate the locations of boundaries between distinct chromatin domains. Light blue vertical line indicates the absence of a clear domain boundary at AU1.

Chromatin looping plays essential roles in gene regulation as it modulates both enhancer-promoter communications and the extent of active or repressed chromatin^16–25,87^. However, high-resolution characterization of chromatin loops, especially in the context of animal development, has been rare^22,88,89^. We have dissected the genomic loops tethered by the SF1 insulator at high resolution to probe their function in the *Drosophila* Hox cluster ANT-C. Our previous studies indicate that SF1 organizes a series of local chromatin loops in the *Scr-ftz* interval to maintain independent regulation of *Scr* and *ftz*^22^. These findings raised further questions on SF1’s functional range of and its level of connectivity. To address these questions, we probed for additional STEs in ANT-C. We identified three distal STEs, LP2, DS1 and AU1 in the *labial*, *deformed* and *Antp* gene regions, extending the SF1’s contact range beyond the local *Scr-ftz* region to include the full extent of ANT-C (red vertical ovals, Fig. 5). Although the new distal STEs bind to diverse insulator proteins they all exhibit enhancer-blocking activity in transgenic assays, an indication that they tether chromatin loops autonomously and independent of their native genomic context. This is in contrast to some proximity STEs located in the *Scr-ftz* region, such as R2 and R6, which do not all contain constitutive enhancer-blocking activity^22^.

Of the three new STEs, both LP2 and DS1 are located near the end of a Hox transcriptional unit, separating the Hox region from a neighboring non-Hox gene region. Both are bound by dCTCF, CP190 and Mod (mdg4), and both insulators colocalize with boundaries between domains of repressive histone marks associated with Hox genes and the less repressive non-Hox domains (red vertical lines, Fig. 5C). In these respects, LP2 and DS1, as well as previously described SF1, SF2 and R10, belong to the “Class I insulators” as defined by the ChIP-seq profile of insulator proteins (orange triangles, Fig. 5)^64^. Intriguingly, out of 13 Class-I insulator protein ChIP sites in the ANT-C intergenic regions, SF1 only captures with four (Fig. 5A,B). This suggests that besides binding of known insulator complexes, other unknown factors may help determining the selectivity of looping events. Further, we showed that DS1 or AU1, when placed in tandem arrangement with SF1, both can mediate enhancer-bypass, whereas LP2 does not. Enhancer-bypass provides evidence that the pairing insulators can interact directly with each other, precluding both partners from looping with other sites. Therefore, inability of the LP2-SF1 pairing to bypass enhancers suggests that the two insulators might not directly contact each other. In this light, their capture in our 3C experiment could result from both elements interacting with a mutual partner simultaneously. Alternatively, since LP2 is the only STE that also interacts with the SuHw insulator protein (Class II), it is possible that the unique combination of insulator complexes interferes with enhancer-bypass (Fig. 5).

The remaining STE, AU1, is bound by GAF, a multi-faceted insulator factor also involved in transcription activation and PcG and Trx-G mediated chromatin organization^65–69,71,90^. As observed above with the Class I insulators, SF1 selectively interacts with R10 and AU1 among multiple other GAF sites present in the ANT-C intergenic regions. AU1 interacts exclusively with GAF and does not clearly demarcate a boundary between distinct histone modification domains (Fig. 5). These suggest that although GAF insulators can tether chromatin loops, mediate enhancer block and enhancer bypass, they do not possess an intrinsic domain boundary or barrier activity^29^. One other major GAF sites in the ANT-C intergenic regions is R10, an STE located at the other end of the ~110-kb *Antp* transcription unit. Together with AU1, the two GAF insulators may play unique roles in modulating *Antp* enhancer traffic, rather than separating silent and active chromatin domains. Previous studies indicate that enhancer-blocking and the barrier function of a boundary element may be mediated by distinct mechanisms^29,76,77^. Our results support the notion that distinct insulator complexes, or combination thereof, dictate unique insulator and boundary behavior and functions.

Using SF1 as the 3C viewpoint, we have now identified a total of six major insulator/boundary elements across the ANT-C Hox complex (Fig. 5A). SF1 selectively captured with these five STEs out of ~20 sites that exhibit similar binding profiles for insulator complexes, suggesting that binding by insulator proteins alone is insufficient to determine looping events. Further, SF1 and STEs occupy all but one of the major domain boundaries between repressive chromatin domains around Hox genes and the less repressive domains around non Hox domains in ANT-C, underscoring the functional relevance of these looping events in organizing the Hox complex in *Drosophila* embryos (Fig. 5B). Use of complementary approaches including transgenic enhancer-block and enhancer-bypass assays provided unique and novel insights on the properties of the STEs and the mechanisms that govern their long-range interactions. Our results highlight the importance of defining chromatin loops in high resolution and with higher specificity. Future work should identify tissue and developmentally regulated factors that dictate the formation of these loops during animal development.

## Materials and Methods

### Chromosome Conformation Capture (3C)

The 3C experiments were performed according to published protocols and as in our previous report^22,51^. All captures were repeated minimally three times (biological replicates). Approximately 3 × 10^7^ nuclei were collected from 0–20 hour old embryos and used in the chromatin preparation according to existing protocols^17,22^. The optimal quantity of template DNA used in PCR reactions was determined empirically by serial dilutions. Briefly, 3C samples were amplified for 20–22 cycles with the outer primer pair (Table S1). Five to ten percent of the outer PCR reaction was amplified with nested inner primers. Capture products were then fractionated on agarose gels and digitally imaged. Quantitation and analysis were done using the Image-J software. All primers were designed to be ~100–150 bp from the restriction site so that all capture products are comparable in size. To generate the control template, purified fly genomic DNA was digested with Eco RI or other restriction enzymes and ligated at a concentration of ~500 ng/μl. The frequencies of capture expressed as Relative Crosslinking, PCR_E_/PCR_C_, is generally plotted over distance^91^. The distance-capture frequency curves were generated using data from ~500 captures between sites with linear distance up to ~200 kb apart, of which the relevant distance range were shown in Fig. 1C. We also generated separate curves for conventional and quantitative PCRs to control for the data range. Statistical analysis and charts were made using the Microsoft Excel and JMP programs. The p-values were calculated using LSMeans Differences Student’s t-test.

### Enhancer-blocking assay in transgenic *Drosophila* embryos

Enhancer-blocking assay, including spacer- and SF1-containing transgenic *Drosophila* lines were described previously^22,34,77,78^. Individual STE sub-fragments were cloned by PCR (see Table S2 for primers), purified after further digestions, and inserted into the Not I site between the NEE and H1 enhancers in pWNHZ vector (Fig. 3A). For transgene containing tandem SF1 and STE, the two insulators were inserted in the same relative position and orientation as in their native genomic loci (Fig. 4A). For transgenes containing LP2, LP2-SF1-ff, LP2-SF1-rf, SF1-SF2-ff, SF2-SF1-ff, and SF1-SF2-fr, genomic integration at the VK33 attP site were mediated by phiC-31 site-specific insertion. For these transgenes, a phiC31 attB site was inserted at the Nsi I site downstream of the mini*white* gene (Fig. 3D,G,J). Microinjections were performed in the Cai lab or by Rainbow Transgene (Camarillo, CA). *In situ* hybridization with *lacZ*, *white* anti-sense RNA probes were performed as previously described^47^. Whole mount *in situ* hybridization and visual assessments of reporter expression were performed according to existing procedures^34,78^. For each pWNHZ transgene, 50–100 embryos were scored double-blindly from at least three independent insertion lines. Briefly, blastoderm stage embryos were scored for *lacZ* level in the NEE domain against the H1 domain (Figs 3B and 4B). Based on the ratio of the *lacZ* in H1/NEE domains, embryos were ranked into four categories from no-block: H/N ≤ 1, weak block: H/N = 2, medium block: H/N = 4, and strong block: H/N ≥ 5.

## Electronic supplementary material

Supplementary Information

## Data Availability

The authors will make all data available upon publication of the manuscript.
