# Supplementary material for: Selective interactions between diverse STEs organize the ANT-C Hox cluster
Source: Sci Rep. 2018 Oct 11;8:15158. doi: 10.1038/s41598-018-33588-4 (PMC6181975; doi:10.1038/s41598-018-33588-4)
Supplement: Supplementary file 1 — Supplementary Information [file 41598_2018_33588_MOESM1_ESM.pdf]

Selective interactions between diverse STEs organize the ANT-C Hox cluster.

Mo Li[&], Zhibo Ma[&], Sharmila Roy, Sapna K Patel, Derrick C. Lane, Carly R. Duffy and Haini N. Cai*

Department of Cellular Biology, University of Georgia, Athens GA 30602

Keywords: insulators, chromatin boundary elements, chromatin loop domains, enhancer-block, enhancer-bypass, homeotic genes, *Drosophila*,

*: Author to whom correspondence should be addressed.

[&]: These authors contributed equally to the study.

Supplemental Information

Supplemental Tables

Table S1 Conventional PCR primers used in SF1 3C capture

Name	Sequence
Antp-0i	CTTTCAGGAATCCCGGGAGTT
Antp-0O	CTACAGCCAGGTCATCACTCA
Antp10i	CTGGGGGATACGAGTGTAAGA
Antp10O	CGAGAGAGGGAAAGAGATCCA
Antp11i	CCCAGCTTAAGTGCTTAACCA
Antp11O	CCAAAGCACTACTACCCCTAAG
Antp12i	CAGCAGCAGTGAAAAACTTGG
Antp12O	ACTATTGGCGTCATCGTGTTG
Antp13i	CAGCTGTACTCAAGTGGTGAT
Antp13O	CGATCTTGGCTAGATTTGAGATCG
Antp1O	ACAAACGCACAAGATGGATGG
Antp2i	TGTTTATAGATTAGCAGAACGTATCTGAG
Antp2O	GTGAAGGAAAACAATTGTACGCA
Antp3i	GCGATCCATTTATCAACGGCA
Antp3O	TTATTGTGCCGCTGCAGATT
antp4i	CCCGCTTATGAAATCGGCATA
Antp4O	GGCACTAAGAGAAGTTTCCAAGA
Antp5i	TCATGGCCACGAAAATGTCCA
Antp5O	GCTCAACTACATCAATGGCCA
Antp6i	GAATCCTTCGCTTTATTACCATGCA

Antp6O	AGGCACAAACAATTTACACACG
Antp7i	GTGGCATCGTTTTTGGTTGTTG
Antp7O	CTTCCCCGCTCAATTGATTCT
Antp8i	TCACAAAAATTACAGGGTGTTGGA
Antp8O	TTATATTTGCAACAGCCAACGGA
Antp9i	CTAGCACGAAGATAACATCACTATTGA
Antp9O	AAGCAATTGTTGAGTAACGGGT
Dfd-5i	CTCACGTGTTTCTGACGTACG
Dfd-5O	AGCAGATGCATCTGCGAAATG
Dfd+5i	TGCAAAAATGACTTAAGGTAGTTAACTT
Dfd+5O	GATTTCCACGGTTCATGCTTT
Dfd+6i	CGCTCATTGTCAACTAATTAGGACA
Dfd+6O	AGTTTCTTAACCCACCTACACG
Dfd1-4fR	AGAAACATTACGATCAGGTTTTACA
Dfd1-4rR	ACGTACATATCTGGTTAAGC
Dfd1-Q	CTTGTTAGCAATTGGAGAGC
Dfd1i	TGGGCTGTGATCAATCATTGG
Dfd1O	AGCTAAGACCAGTTTATCGGAC
Dfd2-Q	CACTTTTCAAATTTCTTCTTACC
Dfd2i	AAACACTGTGCAATGTGCTGA
Dfd2O	AGCTTAATACGTCTGACGTACTTC
Dfd3-Q	CACCTAAATTGGGGTAACTG
Dfd3i	TTAGAACCAAAAGGAGCTGGG

Dfd3O	ATGCGCATATTCCCTTATCGA
Dfd4-Q	AGTTTGCCGGTAATGTTCT
Dfd4i	TGATGTAAATATGTATGTATTTCCCTCC
Dfd4O	CATATGTTCAATTCTGTTGTCCGC
lab-5i	TAAGCTGGAAAACTTTACCTCACC
lab-5O	CTGTCTTTCGAATACCCCAAACC
lab-6i	CAGTGGCGCCTGCATAATAGA
lab-6O	AAACGGACCCCATGTAATGCC
lab-pb1i	GGATTTAATTGTAACTCGAAATCTGGTGA
lab-pb1O	AGATTTCAGGGAGTTTACAGTGGA
lab-pb2i	GACTGTTTTGGTGGAAATTGGCT
lab-pb2O	GTTTTGGTGGTGCTGCTGCTG
lab-pb3i	GGATTAGTTTGAGTTCCGATCAGG
lab-pb3O	TGTCCTTGTTGTCCTCGTCGT
lab-pb4i	TTTCTCCATTTTCGATCCCGAG
lab-pb4O	CACACCCAAAGACCATTTACCT
lab-pb5i	GCCAATGATCTGAACGATGCG
lab-pb5O	TCACGCTCTTTAGAGCCAGAG
lab+4-4F new	GCGGCCGCGAGCGATCACTTTTCGA
lab+4-4R new	GCGGCCGCCGACCAGAAGTGAATTAAATGA
Lab+4-5F	GCGGCCGCTACAACCGAAACTTTCACTT
lab+4-5R	GCGGCCGCACACGTTCAGTGGGTATAA
lab+4-6F	GCGGCCGCTTTTTATACCCACTGAACGT

lab+4-6R	GCGGCCGCCAATTAGGTGTCATCAATGTCA
lab+5i	ATTAACTGCTGTCTTGAGCCG
lab+5O	TAAAAAGGCGAAGGCAGTCTG
labdown 10i	ATGCCCGTCTGATGACGACAA
labdown 10O	GCTTGCAAGAGTCTGAACGAT
labdown 11i	TGATGTCCAAAGCATGTCCTG
labdown 11O	ACATAGTACATTCATGTTGCACGT
labdown 12i	TATTTGAAACAGCTATTACTACAGCTATAGA
labdown 12O	ATACGGTCAGAAACTCTTCCTTCT
labdown 13i	CGTGGAATTTCTGTCCACCAG
labdown 13O	GGAATTGGGCAAATTTAAAAAACCAG
labdown 7i new	GTAAAAGCAAAGCCAGAATGCG
Labdown 7O new	AATCAAACCGATTCCAGGTCG
labdown 8i	AAAATCTCTGAATTAGCCAATATTTTATTTTCATTA
labdown 8O	GCAAGAAATGAAATCGTTATGGGCT
labdown 9i	TGGGTAAATAAAGGTAATAAGATTACTTTTTAACTAT
labdown 9O	TAAGGGCACTCCCCAAAAACT
labdown1i	GGAAGATCAGTGTTATTGCGCT
labdown1O	TTTAAGTGCATGCATGGCCAT
labdown2i	TCCTGTGGCACTTTACTAGAACTT
labdown2O	CGAAAACCAGCTCCTCCTGGT
labdown3i	GTGCATACAGGCGATTGAGAA
labdown3O	GCCGCAGGATTTTGTTTATGG

labdown4i	CAGCCGGGCAAACCGTCTATG
labdown4O	GTAACGACATCAAGGCAAAGGT
labdown5i	GATGTTGGCATCGGTGCTCCA
labdown5O	TTGTCAGGGGATCGACTGTCA
labdown6i	GGGAAAGCTTGATGCAACCAT
labdown6O	AAGGCGTTTTGGGGCATAGTT
labdown7i	CAATTGCACAGAAAAAGTTTCGCT
labdown7O	CGGCTTTTACCCGCTTTTCTT
pb-1inew	CCCCGAACTGTGATAAACGGA
pb-1Onew	GTGCTCATGGTCAACAAGATG
pb-6i	TCTTAGAGCATTCTGAATGAAGGA
pb-6O	TCCTGTGAATATGAGCTTGTTAATGT
pb-Dfd1i	GCAACATCCTGTAAATAAACAGAAATGTATGAA
pb-Dfd1O	TCAATTCAGTCGAGTCCCGAA
pb-Dfd2i	AAATCGGGTTTTTCCTTGCCG
pb-Dfd2O	TTTCGGACCAAAACCTCCCCT
pb-Dfd3i	ACCTACACACTTGGTAGCATAATCA
pb-Dfd3O	TGTGATCCATTCTGTTCTTGGCT
pb-Dfd4i	CACGCGAAATGTCCGCTTACA
pb-Dfd4O	TATTTGAGTATGTAGTCGGCGCA
pb-Dfd5i	TGACTTCTTGGCAGCAATGCA
pb-Dfd5O	GTAAGGATTCCGAGCTCGTTG
pb-Dfd6i	CGCCCTGGATCTGAAGACATA

pb-Dfd6O	CATTATGCACACTTCGCCCGT
pb-Dfd7i	GGATTAACATAAACTGAATGCCTGGA
pb-Dfd7O	GACCACCGTTCAGGACGATAG
pb-Dfd8i	AATACATAAAAACACAAGATATTTAAACACCAATTTATAAT
pb-Dfd8O	CGGCCCAAATAAGGAACTACA
pb+5i	ATCTGTAGGGTTGGCCGTATT
pb+5O	GATACTCAATCCCCATCTCGG
Scr-1i	GGCTGCATGCGATATTAACCA
Scr-1O	CGCTGGCTGACATCTTTTGAT
Scr-2i	GTGCCCGTATCTGACCAACTT
Scr-2O	CTCTACAGAAATGATGTATCTCTTCCG
SF1-1i	CAAAATCGATTTTGGGTGTGTCT
SF1-1O	ACGAATGACCTATGCCATTTATCT
SF1iloop2R	TCTTTCCCTTTAATGTGGCCT
SF1iloop3R	CTGAAATTTCGAAAGTATGAA
SF1iloop5	CCCACACTATGCGTGTGAAC
SF1iloop6	CCGCAGGACGATAATGAACA
SF1iloop7	TAATAACCGACAGCTCGGGT
SF1iloop7R	ACCCGAGCTGTCGGTTATTA
SF1iloop8	TTGCATGTGACGCTAAACGG
SF1iloop8R	CCGTTTAGCGTCACATGCAA
SF1iloop9	TCTTTCCCTTTAATGTGGCCT

Table S2 PCR primers used for cloning STEs in enhancer-blocking transgenes

Name	Sequence
LP2	GCGGCCGCTTTTTATACCCACTGAACGT
	GCGGCCGCCAATTAGGTGTCATCAATGTCA
DS1	TGTAAAACCTGATCGTAATGTTTCT
	GCTTAACCAGATATGTACGT
AU1	GGAATTCTCAGCTAACGGTAGC
	CGCTATTTTAAGAAATTAAAAGCAAATG

Figure S1.

Figure S1. Relative capture frequency of three STE fragments and the surrounding EcoRI fragments in the *labial (lab)* (A), *Deformed (Dfd)* (B) and *Antp (*C) regions. The dashed line in Panel C represents a distance-capture frequency curve [1]. The p-values were calculated using LSMeans Differences Student's t-test.

Figure S2.

Insulator proteins binding profiles at previously identified STEs. Screen crops of the ChIP-Chip track of known insulator proteins in 0-12h embryos surrounding the SF1, SF2 and R10 Map coordinate is based on ModEncode GBrowser dm3 (BDGP R5, (http://www.genome.gov/modENCODE). Color shaded bars represent the cloned STE fragments with STE names on top. Yellow-shaded boxes represent called peaks for bound proteins.

Figure S1

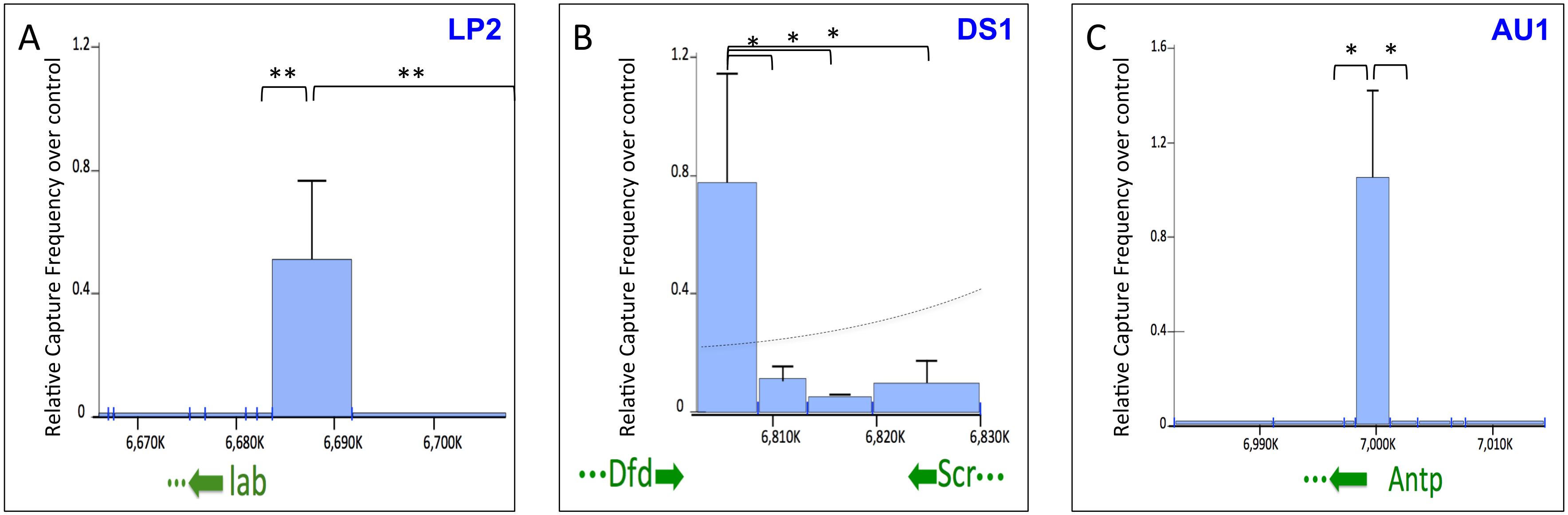

A
LP2
Relative Capture Frequency over control
1.2
0.8
0.4
0
**
**
6,670K
6,680K
6,690K
6,700K
lab
B
DS1
Relative Capture Frequency over control
1.2
0.8
0.4
0
*
*
*
6,810K
6,820K
6,830K
Dfd
Scr
C
AU1
Relative Capture Frequency over control
1.6
1.2
0.8
0.4
0
*
*
6,990K
7,000K
7,010K
Antp

Figure S2

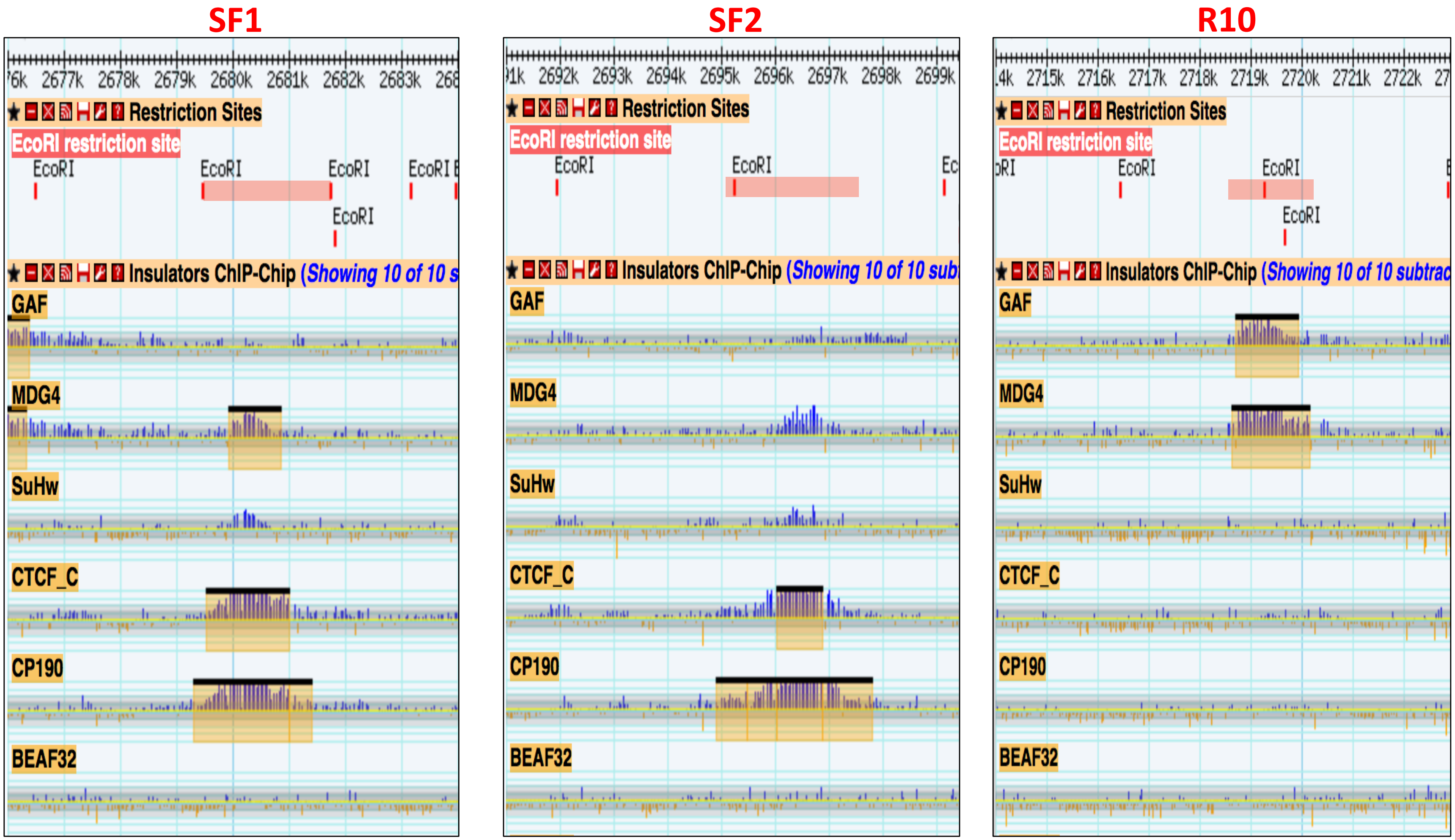
SF1
SF2
R10
Restriction Sites
EcoRI restriction site
EcoRI
Insulators ChIP-Chip (Showing 10 of 10 s
GAF
MDG4
SuHw
CTCF_C
CP190
BEAF32
